# Aging DPP6‐KO mouse showed sleep disorders and seizure related Alzheimer's disease/dementia using *in vivo* EEG detection

**DOI:** 10.1002/alz70855_099362

**Published:** 2025-12-23

**Authors:** Lin Lin, Dax Hoffman

**Affiliations:** ^1^ NIH, Bethesda, MD, USA

## Abstract

**Background:**

We have found multiple novel roles for voltage‐gated potassium channel auxiliary subunit DPP6 (Dipeptidyl Peptidase‐Like 6) including in neuronal development, learning and memory and connections to Alzheimer disease (AD)/dementia. Novel structures associated with amyloid‐β (Aβ) were found in hippocampal area CA1 in aging DPP6‐KO mice, apparently derived from degenerating presynaptic terminals, that are significantly more prevalent in DPP6‐KO mice compared to WT mice. Aging DPP6‐KO mice also show increased Aβ, tau pathologies, neuroinflammation and sleep disturbances.

**Method:**

*in vivo* surgical implantation of the HD‐X02 telemetry system (DSI)

**Result:**

The results showed twelve‐month‐old DPP6‐KO mice have sleep disorders that include more wake and REM and less NREM duration in the light‐on phase. For seizure study, aging DPP6‐KO mice showed significantly increased and longer spike train durations, also exhibited a significantly higher prevalence of spike wave discharges and nonconvulsive seizures with no accompanying tonic‐clonic movements compared to WT controls. We also found increase in the number of epileptiform spike events (over 400 mV) in 12‐month‐old DPP6‐KO mice, and the epileptiform spike events were suppressed after acute administration of anti‐seizure medicine levetiracetam (LEV)

**Conclusion:**

Together these results indicate that DPP6‐KO mice have sleep disorders and seizure with epileptiform events, further supporting a role of DPP6 in Alzheimer's/dementia.

